# Slow motor neurons resist pathological TDP-43 and mediate motor recovery in the rNLS8 model of amyotrophic lateral sclerosis

**DOI:** 10.1186/s40478-022-01373-0

**Published:** 2022-05-14

**Authors:** Seong Kwon Hur, Mandana Hunter, Myrna A. Dominique, Madona Farag, Dejania Cotton-Samuel, Tahiyana Khan, John Q. Trojanowski, Krista J. Spiller, Virginia M.-Y. Lee

**Affiliations:** 1 Center for Neurodegenerative Disease Research, Perelman School of Medicine, University of Pennsylvania, Maloney Building, 3rd Floor, 3600 Spruce Street, Philadelphia, PA 19104-2676 USA; 2 Department of Pathology and Laboratory Medicine, Perelman School of Medicine, University of Pennsylvania, 3400 Civic Center Blvd, Philadelphia, PA 19104 USA; 3 Alzheimer’s Disease Research Center, Perelman School of Medicine, University of Pennsylvania, 3400 Civic Center Blvd, Philadelphia, PA 19104 USA; 4 Janssen Research and Development, Neuroscience Therapeutic Area, 1400 McKean Rd, Spring House, PA 19002 USA

**Keywords:** Neurodegeneration, TDP-43, Amyotrophic lateral sclerosis, Neuropathology, rNLS8, Cross-reinnervation surgery

## Abstract

In the intermediate stages of amyotrophic lateral sclerosis (ALS), surviving motor neurons (MNs) that show intrinsic resistance to TDP-43 proteinopathy can partially compensate for the loss of their more disease-susceptible counterparts. Elucidating the mechanisms of this compensation may reveal approaches for attenuating motor impairment in ALS patients. In the rNLS8 mouse model of ALS-like pathology driven by doxycycline-regulated neuronal expression of human TDP-43 lacking a nuclear localization signal (hTDP-43ΔNLS), slow MNs are more resistant to disease than fast-fatigable (FF) MNs and can mediate recovery following transgene suppression. In the present study, we used a viral tracing strategy to show that these disease-resistant slow MNs sprout to reinnervate motor endplates of adjacent muscle fibers vacated by degenerated FF MNs. Moreover, we found that neuromuscular junctions within fast-twitch skeletal muscle (tibialis anterior, TA) reinnervated by SK3-positive slow MNs acquire resistance to axonal dieback when challenged with a second course of hTDP-43ΔNLS pathology. The selective resistance of reinnervated neuromuscular junctions was specifically induced by the unique pattern of reinnervation following TDP-43-induced neurodegeneration, as recovery from unilateral sciatic nerve crush did not produce motor units resistant to subsequent hTDP-43ΔNLS. Using cross-reinnervation and self-reinnervation surgery in which motor axons are disconnected from their target muscle and reconnected to a new muscle, we show that FF MNs remain hTDP-43ΔNLS-susceptible and slow MNs remain resistant, regardless of which muscle fibers they control. Collectively, these findings demonstrate that MN identity dictates the susceptibility of neuromuscular junctions to TDP-43 pathology and slow MNs can drive recovery of motor systems due to their remarkable resilience to TDP-43-driven degeneration. This study highlights a potential pathway for regaining motor function with ALS pathology in the advent of therapies that halt the underlying neurodegenerative process.

**Supplementary Information:**

The online version contains supplementary material available at 10.1186/s40478-022-01373-0.

## Introduction

Plasticity and functional compensation is commonly observed among surviving neurons following brain or spinal cord injury [1, 29, 33]. While this phenomenon has been principally studied in the context of acute nerve damage, in the early stages of neurodegeneration, disease-resistant neurons can slow symptomatic decline [13, 32, 35]. In amyotrophic lateral sclerosis (ALS), fiber typing in muscle biopsies of patients shows that surviving motor neurons (MNs) expand their motor field early in the disease course [5]. This compensation allows ALS patients to maintain motor function for some time [11, 31], although the rate of MN degeneration eventually overwhelms the ameliorative reinnervation [26, 43]. Furthermore, postmortem samples from the majority of ALS patients have mislocalized TDP-43 in surviving MNs [23, 24, 30, 41] suggesting an intrinsic resistance to TDP-43 pathology in some MN subpopulations. Unfortunately, these cells are inaccessible in early disease stages, making efforts to elucidate the molecular mechanisms by which surviving MNs resist TDP-43 pathology and reinnervate vacated neuromuscular junctions (NMJs) particularly challenging. One solution is the rNLS8 mouse model, in which doxycycline-regulated, neuron-specific expression of human TDP-43 lacking a nuclear localization signal (hTDP-43ΔNLS) results in pathological TDP-43 neuronal inclusions and symptomatic disease that recapitulates clinical ALS [42]. With temporal control of the disease process, this model provides a novel tool to investigate neuronal plasticity at intermediate disease stages. We previously demonstrated that MN subpopulations in rNLS8 mice—such as hypoglossal MNs and fast fatigable (FF) MNs in the lumbar spinal cord (SC)—show selective vulnerability to disease and that surviving MNs can functionally compensate for susceptible MNs, even into late disease stages wherein 40% of all lumbar MNs are lost [37, 40, 42]. However, the mechanism of this compensation and the identity of surviving MNs remain unclear. Accordingly, in the present study, we characterize the disease-resistant, surviving MN subpopulation in the rNLS8 model and the mechanism by which these MNs mediate motor unit expansion, reinnervation and functional motor recovery, as well as how they cope with pathological TDP-43 after they assume new functional connections. We demonstrate that SK3-positive slow MNs are more resistant to TDP-43 proteinopathy than Mmp9-positive FF MNs and can sprout to reinnervate adjacent motor pools vacated by disease-susceptible MNs. Moreover, slow MNs confer resistance to subsequent hTDP-43ΔNLS-triggered axonal dieback from typically susceptible fast skeletal muscles. Using sciatic nerve crush and cross-reinnervation surgery, we show that MN identity rather than muscle fiber type dictates the resistance phenotype and that it is specific to the selective neurodegeneration driven by TDP-43, rather than nerve injury and recovery. These findings identify a potential mechanism by which the function of motor circuits may be restored in ALS patients if therapies that ameliorate the underlying neurodegenerative process can be developed.

## Materials and methods

### Mice

As described previously [37, 42], rNLS8 mice were generated by crossing animals expressing the tetracycline transactivator (tTA) protein under the control of the human *NEFH* promoter with a second line transgenic for human TDP-43 with a defective nuclear localization signal (hTDP-43ΔNLS) under a “tetracycline-off” promoter. In the resulting rNLS8 bigenic mice, dietary doxycycline (Dox Diet #3888, Bio-Serv) inhibits tTA from binding to the tetracycline promoter element, thereby suppressing hTDP-43ΔNLS. Transgene expression was induced by substituting standard chow (Rodent Diet 20 #5053, PicoLab). Male and female rNLS8 mice were randomized to treatment groups at age 2–5 months. Investigators were blinded to the genotype and treatment of each mouse during data collection, and identities were unblinded subsequently for analysis using tattooed identification numbers. Sample sizes were chosen based on power estimates informed by prior publications using the rNLS8 model [37, 40]. All procedures observed the NIH Guide for the Care and Use of Experimental Animals. Studies were approved by the Institutional Animal Care and Use Committee of the University of Pennsylvania.

### Neuronal tracing

To label MNs innervating the tibialis anterior (TA) with GFP before disease onset, P4 pups (*n* = 16) were anesthetized by hypothermia and the TA muscle was injected with 1 μL of AAV9-CMV-PI-eGFP-WPRE-bGH vector (3.3 × 10^13^ gc/mL, Penn Vector Core) using 31-gauge needles with 1/2 cc insulin syringes (BD Ultra-Fine^∗^ II Short Needle Insulin Syringe, VWR). Pups were returned to their mother and their health was monitored to adulthood. Upon reaching adulthood, the mice were randomized between hTDP-43-ΔNLS insult (Dox withdrawn, *n* = 8) and control (maintained on Dox for transgene suppression; *n* = 8) cohorts. After 6 weeks of hTDP-43-ΔNLS expression, doxycycline was reintroduced to allow axonal regeneration to occur. After 8 weeks of recovery, Cholera Toxin Subunit B conjugated to Alexa Fluor 594 (CTB594; 3 µL, Invitrogen) was injected into the same TA muscle. All animals were sacrificed 4 days thereafter, and the lumbar 3–4 regions of the SC were analyzed for tracer labeling of the neuronal soma.

### Retrograde tracing

CTB-594 (3 µL) was injected into the TA of mice as described above to effect retrograde labelling in the L3 level of the lumbar spinal cord of MNs innervating the injected muscle [22]. Four days after CTB-594 administration, the mice were perfused with PBS followed by 4% paraformaldehyde and the lumbar SC removed for analysis of the TA motor pool.

### Muscle physiology

To perform compound muscle action potential (CMAP) recordings, mice were anesthetized using a KAX cocktail (ketamine 60–100 mg/kg, xylazine 8–12 mg/kg, acepromazine 0.5–2 mg/kg), and their hind legs were shaved. The sciatic nerve was stimulated with brief electrical currents applied using bipolar needle electrodes (0.3 Hz, 0.5-ms pulse duration, starting at 0 mA and incrementing by 5 mA). The response from the gastrocnemius (GC) muscle was recorded using needle electrodes placed in the center of the muscle and in the tendon [39]. The M-wave was measured at each amplitude until the maximal response was elicited. Maximum evoked amplitudes were analyzed using the pCLAMP 10 software suite (Molecular Devices).

### Nerve crush

The mice were anesthetized as described above, the lateral thigh of rNLS8 or non-transgenic (nTg) mice was shaved, and a 0.5–1 cm incision in the skin was made over the lateral femur. For the nerve crush, the sciatic nerve was located and crushed with a hemostat for 15 s [6]. The skin incision was closed with silk sutures, and the animals were allowed to recover on a warming blanket.

### Cross-reinnervation surgery

Surgery was performed aseptically following the IACUC Guidelines for rodent survival surgery. The mice were anesthetized as described above, the lateral hindquarters at the incision site were shaved, and a 0.7–1 cm cutaneous incision was made. The fascial plane was opened between the gluteus maximus and the anterior head of the biceps femoris to reveal the common peroneal and tibial nerves. For cross-reinnervation surgery, the nerves were severed, and the proximal stump of the tibial nerve was cross-sutured to the distal stump of the common peroneal nerve via a 3 mm silicon laboratory tubing (0.025 in ID, 0.047 in OD, Fisher Scientific) with 10.0 nylon. The distal stump of the tibial nerve was similarly cross sutured to the proximal stump of the common peroneal nerve. For surgical control, the nerves were exposed and severed, and the proximal stump of the common peroneal and tibial nerves was sutured to the distal stump of corresponding the common peroneal and tibial nerves via a 3 mm silicon laboratory tubing with 10.0 nylon for self-reinnervation surgery. The gluteal musculature was then re-opposed and sutured with 6.0 nylon skin incision. After surgery, mice were maintained under a warming lamp until fully conscious.

### Immunofluorescence

rNLS8 and nTg mice were perfused with ice-cold PBS followed by 4% paraformaldehyde, and the brain, lumbar SC, and hindlimb muscles were collected. Peripheral tissues were washed in PBS overnight, while CNS tissues were fixed in 10% formalin overnight. All tissues were then washed in PBS and processed in a sucrose gradient up to 30% for cryoprotective embedding. To analyze MN populations in the brainstem and lumbar SC, tissues were sectioned at 20 μm thickness, incubated with citric acid (95 °C for 3 min) for antigen retrieval, incubated in 5% FBS in PBS for blocking and immunostained using the following primary antibodies: guinea pig anti-VAChT (1:1000, CNDR, U. Pennsylvania), rabbit anti-VAChT (1:5000, CNDR, U. Pennsylvania), mouse anti-SV2 (2–5 µg/mL, DSHB, U. Iowa), rabbit anti-NFL (1:500, CNDR, U. Pennsylvania), mouse anti-human TDP-43 mAb (0.06 μg/mL, CNDR, U. Pennsylvania, clone 5104), rabbit anti-MMP9 (1:1000, Abcam), goat anti-MMP9 (1:2000, Sigma-Aldrich), rabbit anti-SK3 (Kca2.3, KCa3, Kcnn3, SKCa3) polyclonal (1:500, Millipore), rat anti-phospho-TDP-43^S409−S410^ (1:5000, Cosmo Bio Co.), and mouse anti-pan-TDP-43 (1:10.000, CNDR, U. Pennsylvania). After overnight incubation, tissue sections were washed and then incubated with AlexaFluor secondary antibodies (1:1000, Molecular Probes) for 2 h. Sections were imaged using either a Nikon Eclipse Ni inverted microscope or a Leica TCS SPE confocal microscope. For confocal imaging, 10 z-steps spaced 1–3 µm apart were collected per image, and a maximum projection was created for each. For the quantification, MNs were counted in transverse 20 µm cryosections, 100 µm apart, over a length of 1 mm, using Image J and NIS-Elements software.

The soleus, lateral GC and TA muscles were dissected [13, 32] and sectioned at 30 μm longitudinally, and immunostained for VAChT or SV2 (as described above) to label nerve terminals and treated with α-bungarotoxin conjugated to Alexa Fluor 488 (BTX, 1:1000) to label motor endplates. The proportion of innervated NMJs was scored as the number of areas positive for both SV2 (or VAChT) and BTX (i.e., the total number of innervated endplates) divided by the total number of all BTX-positive areas (i.e., the total number of endplates) on consecutive longitudinal 30 μm cryosections. In this manner, 500–1000 NMJs were scored per TA and lateral GC, and 200–500 NMJs per soleus.

### Muscle fiber typing

rNLS8 and nTg mice were perfused with ice-cold PBS followed by 10% (*v/v*) neutral buffered formalin. The hindlimb muscles were collected, washed in PBS overnight and processed in a 10–30% sucrose gradient for cryoprotective embedding. The muscles were cross-sectioned at 10 µm thickness, incubated in 5% FBS in PBS for blocking, and then immunostained using the following primary antibodies: mouse anti-BA-D5 (for myosin heavy chain type I; 2–5 µg/mL, DSHB, U. Iowa), mouse anti-BF-F3 (for myosin heavy chain type IIB; 2–5 µg/mL, DSHB, U. Iowa), mouse anti-SC-71 (for myosin heavy chain type IIA; 2–5 µg/mL, DSHB, U. Iowa) [36]. After overnight incubation, tissue sections were washed and then incubated with Alexa Fluor secondary antibodies (1:1000, Molecular Probes) for 2 h. Sections were imaged using a Nikon Eclipse Ni inverted microscope.

### TreadScan

Gait analysis was performed using the TreadScan software from the CleverSys NeurodegenScan Suite. The BcamCap image capture system recorded the footprints of the mice locomoting across a transparent treadmill using a high-speed camera at 100 frames per second. The mice were trained on the treadmill at 10–20 cm/s for 20 s periods for 5 training sessions over a 10-d period. The chamber and treadmill were wiped down with 0.25% bleach then with distilled water. After a 3-min habituation on the treadmill, the track speed was slowly increased from 5.0 cm/s until the mouse could no longer keep pace or until 20.0 cm/s was achieved [37, 42]. Instantaneous running speeds and running times were derived from image analysis by the TreadScan software and output for statistical analysis.

### Statistics

Data were first tested for normality (Shapiro–Wilk test) and equivalent variances (Brown-Forsythe test). To determine the statistical significance of comparisons between two groups, unpaired two-tailed t tests were used, except for nerve crush experiments, which used paired two-tailed t tests. One-way ANOVA with the Holm-Sidak method for pairwise multiple comparisons was used when comparing multiple groups. Two-way ANOVA using Tukey’s multiple comparison test was used to evaluate the effect of two factors (e.g., surgical procedure and time post-surgery). Statistical tests were implemented in SigmaPlot and GraphPad Prism 7. *P*-values < 0.05 were considered statistically significant.

## Results

### rNLS8 mice recover motor function and show clearance of cytoplasmic TDP-43 in MNs following hTDP-43ΔNLS suppression

**Fig. 1:**
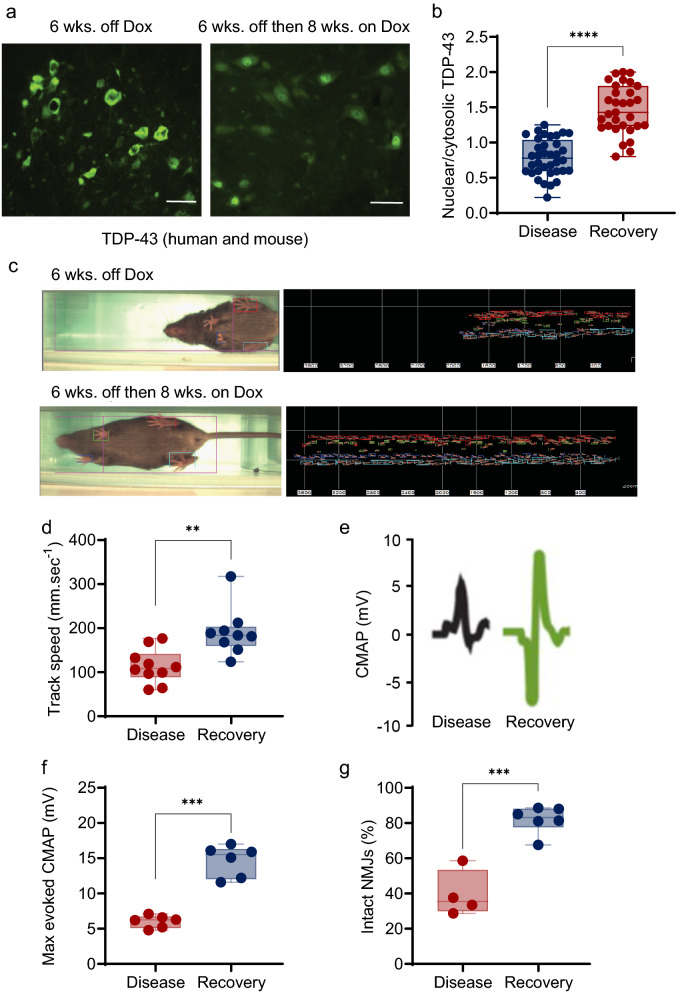
rNLS8 mice recover motor function following hTDP-43ΔNLS suppression after disease onset. **a** Representative immunofluorescence images of rNLS8 lumbar MNs expressing high levels of cytoplasmic TDP-43 at 6 wks. off Dox compared to low levels of cytoplasmic TDP-43 after 8 wks. recovery following 6 wks. off Dox. Scale bar: 50 μm. The antibody used for immunostaining detects both human and murine TDP-43. **b** Comparison of immunofluorescence staining intensity for TDP-43 as shown in (**a**) in the nucleus and cytoplasm of lumbar MNs from rNLS8 mice at 6 wks. off Dox or 6 wks. off Dox followed by 6–8 recovery on Dox. The nuclear area was defined by reference to DAPI staining and the nuclear/cytosolic TDP-43 immunofluorescence intensity ratio was calculated for 40–50 cells per group. The latter metric was compared between groups by unpaired t test; ****p < 0.0001. **c** TreadScan gait analysis with stance graph for representative rNLS8 mice at 6 wks. off Dox compared to 6 wks. off Dox followed by after 8 wks. recovery on Dox. **d** Treadmill running speeds of rNLS8 mice after 6 wks. off Dox and after 6 wks. off Dox followed by 6–8 wks. recovery on Dox. N = 9 mice per group, t test; **p = 0.002. **e** Average traces of compound muscle action potential (CMAP) in the gastrocnemius (GC) muscle of rNLS8 mice after 6 wks. off Dox or 6 wks. off Dox followed by 6–8 wks. recovery on Dox. **f** Maximum CMAP amplitude in the GC muscle of rNLS8 mice after 6 wks. off Dox or 6 wks. off Dox followed by 10 wks. recovery on Dox (t test, ***p < 0.001). **g** The proportion of neuromuscular junctions in the fast hindlimb muscle tibialis anterior (TA) innervation in rNLS8 mice after 6 wks. off Dox or 6 wks off Dox followed by 6–8 wks. recovery on Dox (t test; ***p = 0.002)

We have previously reported that fast fatigable (FF) MNs are selectively vulnerable to disease in rNLS8 mice [37]. The fact that motor symptoms resolve upon suppression of the hTDP-43ΔNLS transgene by reintroducing dietary Dox after disease onset (i.e., after axonal dieback [11] of FF MNs has already occurred) suggests that surviving, disease-resistant MN subpopulations exhibit plasticity and compensation in the rNLS8 model [37, 40, 42]. To explore this compensation, we characterized motor recovery in rNLS8 mice via functional, electrophysiological, and histological investigations. First, we confirmed that reintroducing dietary Dox after disease onset in rNLS8 mice cleared TDP-43 expression in the cytosol and restored endogenous TDP-43 in the nucleus of MNs in the lumbar SC (Fig. 1a, b). Then, using TreadScan software to compare the locomotion of rNLS8 mice after 6 weeks of hTDP-43ΔNLS expression (corresponding to late disease in this model) with the same animals after a subsequent 6–10-wk. recovery period on Dox (Fig. 1c), we observed a significant improvement in the average instantaneous running speeds (Fig. 1d). To examine the electrophysiological response to motor nerve stimulation in these mice, we recorded the evoked compound muscle action potentials (CMAP) in the gastrocnemius (GC) muscle (Fig. 1e) and found a significant increase in the peak amplitudes of the maximum M-wave at 10 wks. after transgene suppression (Fig. 1f), suggesting reinnervation of neuromuscular junctions (NMJs) within the GC. We confirmed NMJ reinnervation morphologically in another fast-twitch muscle—the tibialis anterior (TA)—by staining for the synaptic vesicle glycoprotein (SV2) and with fluorophore-conjugated alpha-bungarotoxin (BTX) to label motor terminals and endplates, respectively. By scoring the fraction of total BTX-positive endplates that were coincident with SV2-positive motor terminals (i.e., innervated), we found significant reinnervation to occur after 6 weeks of transgene suppression (Fig. 1g). These data established that the recovery of motor function following hTDP-43ΔNLS transgene suppression in rNLS8 mice is concurrent with the reinnervation of motor units to susceptible fast-twitch muscles denervated during the initial ALS-like disease course.

### Intrinsically disease-resistant MNs expand to adjacent fields to reinnervate susceptible muscle after the suppression of hTDP-43ΔNLS in rNLS8 mice

**Fig. 2:**
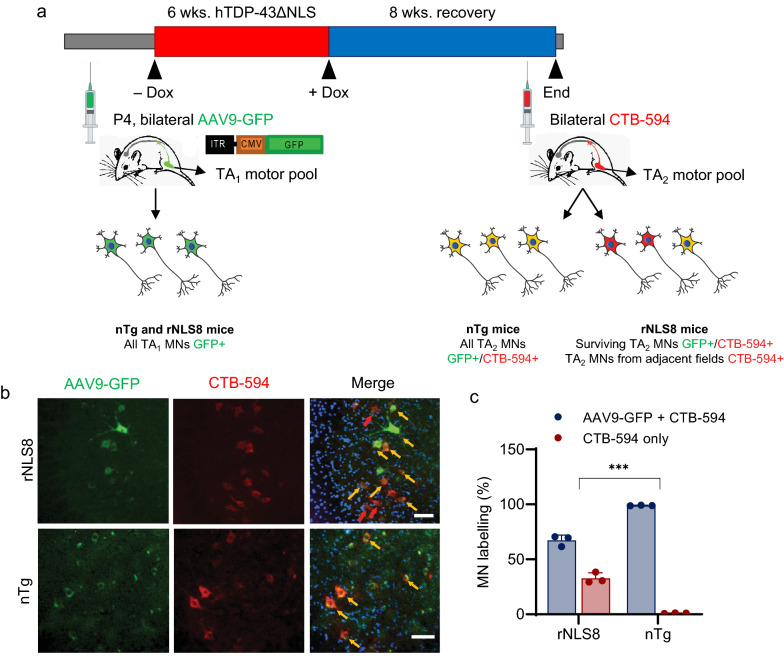
Intrinsically disease-resistant MN subpopulations mediate the reinnervation of vulnerable muscles after disease onset in rNLS8 mice. **a** The experimental approach used to investigate the identity of MNs that reinnervate the TA muscle during recovery in rNLS8 mice. The retrograde neuronal tracer AAV9-GFP was injected bilaterally in the TA of p4 mice to fluorescently label (in green) the pre-disease motor pool (denoted TA_1_). Dox was then withdrawn for 6 wks. to activate hTDP-43ΔNLS expression, followed by 8 wks. Dox reintroduction for recovery, after which CTG-594 was injected bilaterally in the TA to fluorescently label (in red) the post-disease motor pool (denoted TA_2_). Surviving local MNs would thus be labelled with both green and red fluorescence, whereas MNs from adjacent pools expanding their field to reinnervate the TA would be labelled with red fluorescence only. Below are illustrated potential outcomes for the experiment, including the interpretation of TA_2_ MNs that were either exclusively co-labelled with GFP and CTB-594 or were labelled with CTB-594 alone. **b** Representative images of lumbar MNs co-labeled with AAV9-GFP and CTB-594 from rNLS8 and non-transgenic (nTg) mice at the endpoint of the experiment outlined in **a**. Yellow arrows mark MNs labelled with both AAV9-GFP and CTB-594 (i.e., surviving local MNs), while red arrows mark MNs labelled with CTB-594, only (i.e., MNs reinnervating from adjacent motor pools). Scale bar: 30 µm. **c** Quantification of the proportion of MNs labelled with CTB-594 only (i.e., MNs reinnervating from adjacent motor pools) or both AAV9-GFP and CTB-594 (i.e., surviving local MNs) in lumbar sections from rNLS8 and nTg mice at the endpoint of the experiment outlined in **a**. Three mice were examined per group, with 20–30 MNs scored per animal. T test, ***p = 0.0003

To identify the MN subpopulations that mediate the reinnervation of vulnerable muscles after disease onset, we employed a GFP-expressing adeno-associated virus vector (AAV9-GFP) [20] and Cholera toxin subunit B (CTB) conjugated to Alexa Fluor 594 (CTB-594) as neuronal tracers to identify the cell bodies of MNs innervating the TA muscle [15, 38] before and after hTDP-43ΔNLS insult. Specifically, we injected AAV9-GFP into the TA of rNLS8 or non-transgenic (nTg) pups (P4), such that AAV9-GFP would be retrogradely transported from the peripheral motor axons [19, 20] to label the pre-disease TA motor pool (denoted “TA_1_”). At age 2–5 months, hTDP-43ΔNLS was induced for 6 wks. to elicit axonal dieback, followed by a period of transgene suppression to permit reinnervation of the TA. When the mice exhibited motor recovery, we injected CTB-594 into the TA to label the post-recovery motor pool denoted (“TA_2_”) and thereby identify the compensating MNs (Fig. 2a). We then compared the proportion of MNs in the retrogradely labeled pool that were positive for both AAV9-GFP and CTB-594 (i.e., surviving MNs innervated from the TA_1_ pool) or with CTB-594 only (innervated from adjacent pools; Fig. 2b). While all TA_2_ MNs in the TA of nTg mice were positive for both AAV9-GFP and CTB-594, only 35% of MNs in the TA_2_ pool of rNLS8 mice were positive for CTB-594, suggesting that MNs from another pool, other than the original TA_1_ set, mediate reinnervation of this muscle (Fig. 2b, c).

### Reinnervated motor units are resistant to subsequent hTDP-43ΔNLS insult

**Fig. 3:**
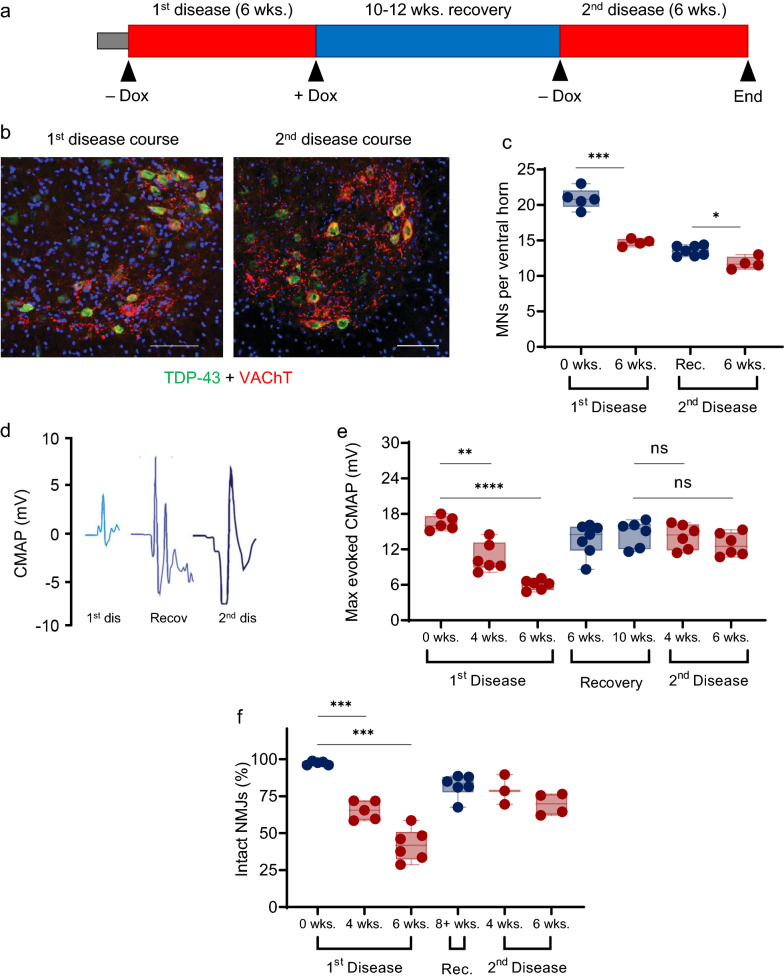
The recovered lower motor circuit in rNLS8 mice is resistant to subsequent hTDP-43ΔNLS challenge. **a** The experimental approach used to investigate the response of rNLS8 mice to a second instance of hTDP-43ΔNLS expression after recovery from the initial pathological insult. Dox was withdrawn for 6 wks. to initiate a first disease course, after which hTDP-43ΔNLS was suppressed for 10–12 wks. by reintroducing dietary Dox, thus allowing muscle reinnervation by resistant MNs. A second 6-wk. instance of disease was then imposed by withdrawing Dox. Arrows indicate the time points at which mice were analyzed for CMAP, TA innervation and MN counts. **b** Representative immunofluorescence images of lumbar MNs (VAChT-positive, red) expressing high levels of cytoplasmic hTDP-43 (green) from rNLS8 mice in the first and second disease courses. Scale bar: 100 μm. **c** Quantitation of the number of MNs per ventral horn in rNLS8 mice at each analysis timepoint in the study outlined in **a**. The number of animals assayed per timepoint was 4–5, with 10–20 spinal cord sections scored for each animal. T test, ***p < 0.001 for baseline vs. 1^st^ disease course; *p = 0.02 for recovery versus 2nd disease course. **d** CMAP traces in the GC muscle from rNLS8 mice after the first disease course, after recovery and after the second disease course. **e** Longitudinal comparison of maximum evoked CMAP amplitude in the GC muscle of rNLS8 mice throughout the experimental time course outlined in (**a**). The number of animals assayed per timepoint was 5–7. T test, **p = 0.0010 for baseline versus 4 wks. off Dox; ****p < 0.0001 for baseline versus 6 wks. off Dox; other comparisons nonsignificant. **f** The proportion of intact NMJs in the lateral GC muscle of rNLS8 mice throughout the experimental time course outlined in (**a**). The number of animals assayed per timepoint was 3–6, with 600–1000 NMJs scored per animal. T test, ***p < 0.001 for baseline versus 4 wks. off Dox; ***p < 0.001 for baseline vs. 6 wks. off Dox; other comparisons nonsignificant

We next investigated whether motor end plates reinnervated by adjacent, ostensibly disease-resistant MNs would inherit this resistance when subjected to a subsequent hTDP-43ΔNLS insult. To test this, after 6 wks. of initial hTDP-43-ΔNLS challenge followed by 10–12 wks. of recovery, we again removed dietary Dox to impose a second hTDP-43ΔNLS disease course and examined the functional and morphological response of the TA_2_ motor pool (Fig. 3a). Strong expression of hTDP-43-ΔNLS in TA_2_ MNs during the second disease course was observed (Fig. 3b), and yet these MNs remained resistant to degeneration when re-challenged with hTDP-43ΔNLS, despite the expansion of the size of individual motor units. Specifically, in contrast to 28.8% of ventral horn MNs lost during the first disease course, only 7.6% of MNs were lost after the second hTDP-43ΔNLS challenge (Fig. 3c). Notably, there was also no decline in maximum evoked CMAP during the hTDP-43-ΔNLS rechallenge (Fig. 3d, e). This finding was recapitulated morphologically, as the initial hTDP-43-ΔNLS insult precipitated a decline in the proportion of intact NMJs in the lateral GC (assessed via SV2 and BTX labelling), while reinnervation was observed during recovery and the reinnervated motor units remained intact during hTDP-43-ΔNLS rechallenge (Fig. 3f).

Next, Mmp9 [18, 27] and SK3 [10, 12] immunofluorescence was performed to identify FF MNs and slow MNs, respectively, and thereby investigate whether these MN subpopulations show differential responses to hTDP-43-ΔNLS rechallenge (Additional file 1: Fig. S1a, b).1: Fig. S1c, d) despite a robust decrease in the ratio of nuclear to cytoplasmic TDP-43 (Additional file 1: Fig. S1e). These data demonstrated that the MNs responsible for reinnervation remain resistant to axonal dieback, suggesting that their intrinsic resistance mechanisms persist despite innervating susceptible, fast skeletal muscles.

### Phenotypic changes induced by nonspecific injury do not drive the resistance of reinnervated motor units to hTDP-43ΔNLS disease

**Fig. 4:**
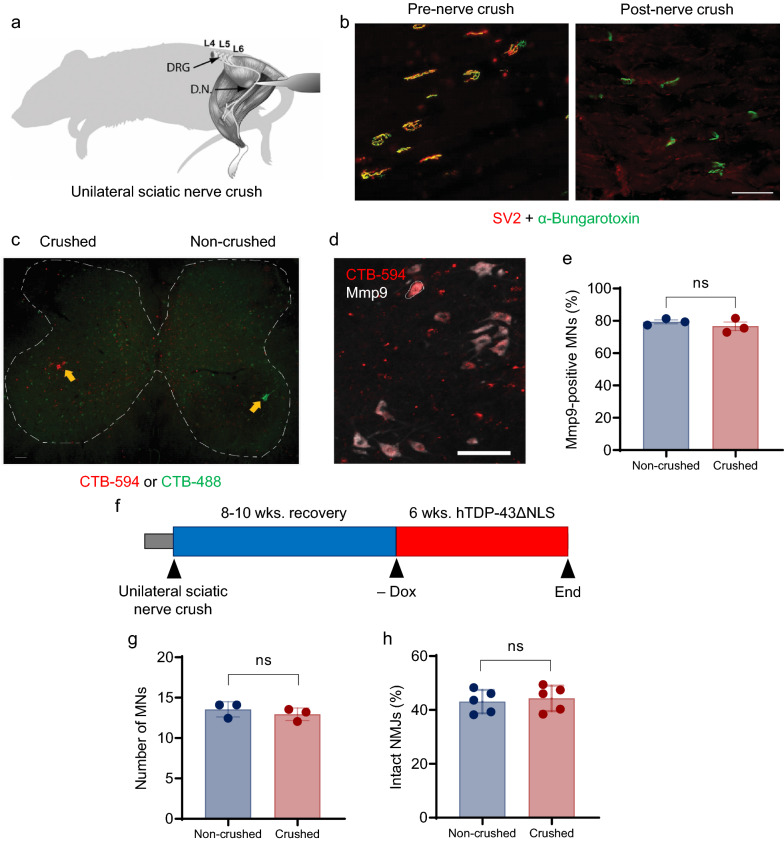
Nonselective degeneration and reinnervation does not alter the susceptibility of the TA muscle to axonal dieback in the presence of pathological hTDP-43ΔNLS. **a** Schematic representation of a unilateral sciatic nerve crush. *DRG* dorsal root ganglion, *DN* distal nerve. **b** Representative fluorescence images of the TA muscle of nTg mice before and after nerve crush. Immunostaining for SV2 marks nerve endings (red), while labelling with BTX-488 marks motor endplates (green). Scale bar: 100 µm. **c** Representative image of TA-innervating MNs in the lumbar spinal cord of nTg mice backfilled with CTB-594 on the crushed side (left, red fluorescence) and with CTB-488 on the contralateral side (right, green fluorescence). Yellow arrows mark MNs reinnervating (crushed side) or innervating (non-crushed) motor pools in the TA. Scale bar: 100 µm. **d** Representative immunofluorescence staining for Mmp9, a marker of fast-fatigable (FF) MNs, in TA-reinnervating MNs (i.e., CTB-594 back-filled; red) on the ipsilateral side of the lumbar spinal cord of nTg mice 10 wks. after unilateral sciatic nerve crush. Scale bar: 100 µm. **e** Quantification of the proportion of reinnervated FF type MNs (Mmp9-positive) among all CTB-488/CTB-594-positive TA MNs on the ipsilateral and contralateral sides of nTg mice subjected to unilateral sciatic nerve crush. Three animals were assayed, with 30–40 MNs scored per animal. Paired t test, n. s., *p* = 0.2049. **f** The experimental approach used to evaluate whether recovery after unilateral sciatic nerve crush in rNLS8 mice ameliorates denervation during subsequent hTDP-43ΔNLS expression. Mice were subjected to unilateral sciatic nerve crush as illustrated in (**a**), then allowed to recover on Dox for 8–10 wks. Bilateral CMAP and TreadScan analysis were used to confirm full recovery, after which hTDP-43ΔNLS expression was induced by withdrawing Dox for 6 wks. MNs were counted and NMJs analyzed bilaterally at endpoint. **g** Quantitation of the number of MNs labelled with VaChT per ventral horn in rNLS8 mice analyzed at endpoint in the experiment outlined in (**f**). Three mice were assayed, with 10 spinal cord sections scored per animal. Paired t test, n. s., *p* = 0.054 **h** Quantitation of the proportion of intact NMJs (scored as coincident SV2-positive nerve endings and BTX-488/BTX-594-labeled motor endplates) relative to all BTX-488/BTX-594-labeled motor endplates in the TA muscles on the ipsilateral and contralateral sides of rNLS8 mice at the endpoint of the experiment outlined in (**f**). Five mice were assayed, with 600–1000 NMJs scored per animal. Paired t test, n. s., *p* = 0.391

We next explored whether the resistance of motor units to hTDP-43-ΔNLS rechallenge is an intrinsic property of MN subtype or arises from injury-induced phenotypic changes. To address this question, we performed unilateral sciatic nerve crush in rNLS8 mice and observed general axonal dieback from NMJs in the TA of non-transgenic mouse (Fig. 4a, b). To identify the MNs that reinnervated the motor endplates that were vacated after the nerve crush, we performed retrograde labeling with CTB-594 in the ipsilateral (crushed) TA and with CTB-488 in the contralateral (non-crushed) TA (Fig. 4c). The mice were allowed to recover for 8–10 wks. and motor and electrophysiological recovery was confirmed. We found that the reinnervated MNs in the fast-twitch TA were positive for the FF MN marker Mmp9, indicating that motor unit identity is maintained (Fig. 4d). Furthermore, we confirmed that the proportion of Mmp9-positive FF MNs reinnervating the TA on the crushed side was indistinguishable from the populations innervating the TA on the non-crushed side (Fig. 4e). We then investigated whether these reinnervated FF MNs acquire resistance to the hTDP-43-ΔNLS insult imposed after recovery from unilateral sciatic nerve crush in rNLS8 mice (Fig. 4f) in the same manner observed for MNs that survive an initial hTDP-43-ΔNLS insult. There was no difference observed in the number of total VAChT-positive MNs per ventral horn on the crushed and non-crushed sides following 6 wks. of hTDP-43-ΔNLS expression (Fig. 4g), and the proportion of intact NMJs was equivalent in the crushed and non-crushed TA muscles (Fig. 4h). These data indicated that reinnervation by FF MNs following a sciatic nerve crush does not alter the susceptibility of the TA muscle to axonal dieback in the presence of pathological hTDP-43ΔNLS. The resistance of motor units to hTDP-43-ΔNLS insult was therefore not driven by generic injury-induced phenotypic changes in surviving MNs.

### Cross-reinnervation surgery substitutes slow-type for FF MN pools in TA motor units and reveals that the axonal regeneration of slow MNs is faster than FF MNs

**Fig. 5:**
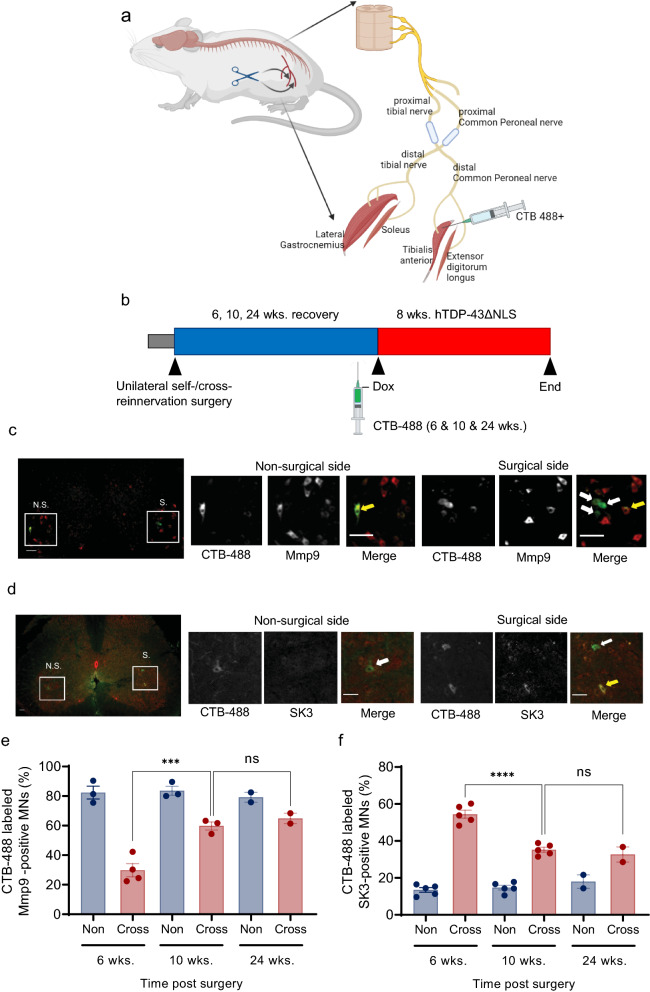
Cross-reinnervation surgery manipulates slow MNs to innervate TA muscle in place of FF MNs. **a** Schematic representation of unilateral cross reinnervation surgery. **b** The experimental approach for using cross-reinnervation surgery to investigate the significance of MN identity in axonal regeneration following hTDP-43ΔNLS disease. rNLS8 mice were subjected to unilateral cross-innervation or self-innervation surgery (the latter as a procedural control) and allowed to recover for 6, 10 and 24 wks. Recovery was confirmed using bilateral TA muscle CMAP and TreadScan analysis. At 6, 10 and 24 wks. post-operation, the TA muscle was bilaterally injected with CTB-488 3 days before sacrificed mice to validate the reinnervation surgery. At 10 wks. post-operation, Dox was withdrawn to activate hTDP-43ΔNLS expression for a period of 8 wks. **c** Representative fluorescence images of sections from the lumbar spinal cord highlighting the ipsilateral (cross-reinnervation surgery; “S”) and contralateral (non-surgical; “N.S.”) sides of rNLS8 mice 10 wks. after surgery. Visible are FF MNs positive for Mmp9 immunostaining (red) and re-innervated TA MNs retrogradely labelled with CTB-488 (green). Mmp9-negative/CTB-488-positive MNs were either slow type or fatigue-resistant TA MNs (white arrows), whereas Mmp9-positive/CTB-488-positive MNs were fast-fatigable TA MNs (yellow arrows). Scale bar: 50 µm. **d** Representative fluorescence images of sections from the lumbar spinal cord highlighting the ipsilateral (cross-reinnervation surgery; “S”) and contralateral (non-surgical; “N.S.”) sides of rNLS8 mice 10 wks. after surgery. Visible are MNs positive for SK3 immunostaining (red) and re-innervated TA MNs retrogradely labelled with CTB-488 (green). White arrows mark SK3-negative, CTB-488-positive presumptive FF TA MNs. The yellow arrow marks as SK3-positive, CTB-488-positive slow type or fatigue-resistant TA MN. Scale bar: 50 µm. **e** Quantification of reinnervated FF type TA MNs (Mmp9-positive/CTB-488-positive) as a proportion of total re-innervated TA MNs (all CTB-488-positive) across the study time course outlined in (**b**). Two-to-four mice were assayed per time point, with 70–80 MNs scored per side. Two-way ANOVA, ***p = 0.0003. **f** Quantification of reinnervated slow type TA MNs (SK3-positive/CTB-488-positive MNs) as a proportion of total re-innervated TA MNs (all CTB-488-positive) across the study time course outlined in (**b**). Three-to-five mice were assayed per timepoint, with 70–80 MNs scored per side. Two-way ANOVA, ****p < 0.0001

To address the question of whether the intrinsic resistance of reinnervated motor units is driven by slow MNs, we performed unilateral cross-reinnervation surgery [14, 16, 25], by which two proximal nerves derived from the sciatic nerve—the common peroneal and tibial nerves—were substituted for two distal nerves connected at two sides of the lateral GC, soleus, TA and exterior digitorum longus muscles (Fig. 5a). Via this procedure, slow MNs were thus experimentally manipulated to innervate fast-twitch muscles. To demonstrate successful cross-reinnervation, rNLS8 mice were subjected to surgery, allowed to recover for 6–24 wks. then CTB-488 was injected into TA to retrogradely label MNs innervating this muscle. Dietary Dox was then withdrawn to activate an 8-wk. TDP-43-driven disease course (Fig. 5b). A self-reinnervation surgery cohort was included as a procedural control for any phenotypic effects resulting from the manipulation. Mmp9 and SK3 immunostaining was performed to verify whether the subtype of retrogradely labeled MNs was altered from FF to slow after cross-reinnervation surgery. We observed that the proportion of Mmp9-positive FF MNs on the cross-reinnervation surgery side was significantly lower than on the contralateral (non-surgical) or self-reinnervation surgery sides (Fig. 5c; Additional file 1: Fig. S2a). Correspondingly, the proportion of SK3-positive slow-type MNs was increased on the cross-reinnervation surgery side relative to the non-surgical or self-reinnervation surgery sides (Fig. 5d; Additional file 1: Fig. S2b). We then performed fiber typing to investigate phenotypic changes in the soleus muscle subjected to cross-reinnervation surgery. We found that the proportion of fast-fatigable fibers positive for myosin heavy chain type IIB was significantly increased by cross-reinnervation surgery (Additional file 1: Fig. S3a–c). These data confirmed that the MNs pools of soleus motor units were successfully substituted after cross-reinnervation surgery. We also compared the kinetics of reinnervation by FF and slow MNs by immunostaining for Mmp9 and SK3 on the non-surgical and cross-reinnervation surgery sides of rNLS8 mice at 6, 10 and 24 wks. post-operative recovery. We found that slow-type MNs reinnervated muscle fiber more rapidly than FF-type MNs, with the greatest surgery-induced changes observed at 6 wks. and 10 wks. post-surgery, respectively (Fig. 5e, f). This observation indicated that slow MNs reinnervate faster than FF MNs and could mediate functional recovery in motor function observed by 6 wks. after surgery (Additional file 1: Fig. S3d).

### Motor unit resistance to hTDP-43ΔNLS is driven by slow MNs

**Fig. 6:**
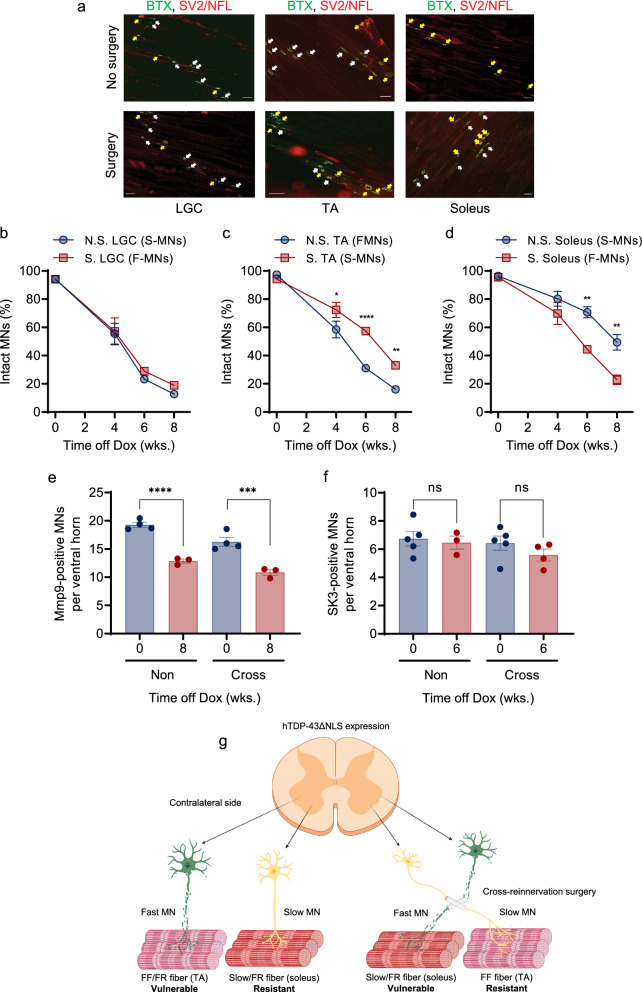
Fast muscle innervated by slow motor units via cross-reinnervation surgery acquire resistance to hTDP-43ΔNLS insult, while slow muscle innervated by fast motor units become susceptible. **a** Representative fluorescence images of NMJs in the hindlimb lateral GC, TA and soleus muscles on the non-surgical and cross-reinnervation surgery sides of rNLS8 mice after 6 wks. off Dox. Axons were immunostained for SV2 and NFL (red) and motor endplates were labelled with BTX-488 (green). Yellow arrows mark intact NMJs (coincident red and green fluorescence), whereas white arrows mark degenerated NMJs. Scale bar: 50 µm. **b**–**d** Quantitation of the proportion of NMJs determined to be intact, by fluorescence image analysis as shown in **a**, in the lateral GC (**b**; two-way ANOVA, n. s. at each time point), TA (**c**; two-way ANOVA, *p = 0.019 at 4 wks. off Dox, ****p < 0.0001 at 6 wks. off Dox, **p = 0.0038 at 8 wks. off Dox) and soleus (**d**; two-way ANOVA, **p = 0.0032 at 6 wks. off Dox; **p = 0.0028 at 8 wks. off Dox) from the cross-reinnervation surgery (S) or non-surgical (N.S.) sides of rNLS8 at the indicated time points after hTDP-43ΔNLS induction. **e**, **f** Quantification of Mmp9-positive fast MNs (**e**; one-way ANOVA, ****p < 0.0001; ***p < 0.001) and SK3-positive slow MNs (**f**; one-way ANOVA, n. s.) in the lumbar region 3–5 on the non-surgical and surgical sides of rNLS8 mice before and after 8 wks. **e** or 6 wks. **f** of hTDP-43ΔNLS expression. **g** Schematic representation of the principal conclusions from the cross-reinnervation surgery study. SK3-positive slow MNs are resistant to degeneration during hTDP-43ΔNLS insult despite being experimentally forced to reinnervate the fast type, vulnerable TA muscle. Mmp9-positive fast MNs are susceptible to TDP-43 pathology despite being experimentally forced to reinnervate the slow type, resistant soleus muscle

We next investigated the response of motor units in muscle subjected to cross-reinnervation surgery or no surgery to hTDP-43ΔNLS challenge. SV2 and NFL immunostaining of nerve terminals with BTX labelling of motor endplates was used to score intact NMJs in the lateral GC, TA, and soleus muscles on the surgical and non-surgical sides of rNLS8 after 0, 4, 6, and 8 wks. of hTDP-43-ΔNLS expression (Fig. 6a–d). The rate of NMJ denervation in the lateral GC showed no difference between the surgical and non-surgical sides (i.e., innervated by FF MNs on both sides; Fig. 6b). However, the proportion of intact NMJs deteriorated more rapidly in the non-surgical TA (innervated by FF MNs) than in the TA surgically cross-reinnervated by slow MNs (Fig. 6b), while the rate of NMJ denervation was greater in the soleus surgically cross-reinnervated by FF MNs than in the non-surgical soleus (innervated by slow MNs).

Finally, to investigate which subtype of MNs was more susceptible to pathological hTDP-43ΔNLS with and without cross-reinnervation surgery, we scored Mmp9-positive FF and SK3-positive slow MNs immediately before and after 6 wks. of hTDP-43-ΔNLS expression. Cross-reinnervated FF and slow MNs both expressed pathological, phosphorylated TDP-43 (Additional file 1: Fig. S4a). However, the number of FF MNs in the lumbar region 3–5 declined significantly during disease on both the contralateral and cross-reinnervation surgery sides (Additional file 1: Fig. S4b, c; Fig. 6e). In contrast, the number of slow MNs was unaltered by pathological TDP-43 on both the contralateral and surgical sides (Additional file 1: Fig. S4d, e; Fig. 6f). These findings established that slow MN identity determines the resistance of motor units to pathological hTDP-43ΔNLS, while FF MN identity dictates the selective vulnerability of motor units to ALS-like disease in rNLS8 mice.

## Discussion

Compensatory neuroplasticity is difficult to study in chronic models of neurodegenerative diseases, at least in part because the selectively resistant neurons are inaccessible in patients and animal models during the time when compensation could be occurring. In the present study, we overcame this challenge by using the rNLS8 mouse model of sporadic ALS, which permits temporal regulation of the TDP-43-triggered degenerative and regenerative cycles. As a model driven by the accumulation of cytoplasmic, hyper-phosphorylated, wild type TDP-43, rNLS8 recapitulates the cellular pathology of most clinical ALS presentations [42]. Moreover, a recent study elegantly demonstrated a mechanism of TDP-43-driven axonal dieback from vulnerable muscles that is operative in tissue from ALS patients, human iPSC-derived motor neurons from an ALS patient and in rNLS8 mice [2]. Though this study implicated mitochondrial function, the nature of iPSC modeling means that long-range neuronal circuits cannot be studied in this system. The rNLS8 model, therefore, provides a valuable tool for investigating functional compensation from motor neurons that survive TDP-43 insult. Using cross-reinnervation surgery and nerve crush experiments, we made the surprising observation that SK3-positive slow MNs reinnervate vacated NMJs in susceptible fast skeletal muscle relatively quickly, forming new motor units that resist degeneration when challenged with a second course of hTDP43ΔNLS expression. In this manner, formerly vulnerable hindlimb muscles acquired resistance to further degeneration. This acquired resistance was specific to TDP-43 insult and driven by MN identity rather than the size of the innervation field or injury-induced (i.e., adaptive) changes in MN phenotype, as sciatic nerve crush did not alter responses to hTDP43ΔNLS rechallenge in an analogous manner.

A clear finding from our study is that slow and FF MNs in the same region of the SC have markedly different intrinsic susceptibility to TDP-43 pathology in rNLS8 mice. Identifying the molecular mechanisms underlying this differential sensitivity would be of high interest from a therapeutic discovery standpoint. Omic analyses could be performed on analytes isolated from FF MNs in the TA and soleus muscles before hTDP43ΔNLS transgene expression and from slow MNs in the same muscles after hTDP43ΔNLS suppression and NMJ reinnervation, enabling feature contrasts between the original, susceptible motor units and the re-wired, resistant motor units. Functional investigation of differentially expressed molecular features could conceivably identify approaches for augmenting the resistance to MNs to TDP-43-driven degeneration. Previous studies have addressed differences in MN susceptibility [8, 17, 31], but these invariably compared different motor pools (for example, the MNs innervating eye muscles compared to leg muscles). As a result, many of the identified differences between MNs could be unrelated to their ability to cope with disease processes, but rather related to developmental patterning, rostrocaudal identity, etc. Further, those previous studies looked at differences between MNs in non-disease contexts or in response to genetic mutations that are rare in clinical ALS [34]. Given the results presented herein, we propose that the rNLS8 mice are a valuable tool to examine MNs that are performing the same function in the same region of the SC, but with divergent responses to the dominant pathological hallmarks of ALS. Understanding the dynamic changes that take place within a defined motor circuit could greatly benefit the future design of drugs with the mechanistic intent of rendering MNs more durable against to TDP-43 pathology.

Beyond characterizing rNLS8 mice as a model for selective resistance to TDP-43, our study outlines a potential mechanism by which ALS patients could hypothetically regain lost motor function in the advent of therapeutic interventions that successfully halt the underlying neurodegenerative process. In this regard, we note that there has been great progress in the last decade in developing tools to target the CNS for gene delivery, knockdown, and editing. New targets that were once considered “undruggable” can now be modulated clinically, and our understanding of the pathological underpinnings of ALS continues to evolve [7, 9, 21]. Currently, multiple academic and industry groups have programs in development targeting genetic mutations known to cause ALS [3], allowing for a future in which the ALS population may be segmented into many subgroups and individually treated by “precision medicine” approaches. For example, ALS patients have recently been administered investigational antisense oligonucleotides (ASOs) that bind to *SOD1* RNA (Tofersen, NCT02623699), transcripts containing the G4C2 repeat expansions of the *C9orf72* gene (BIIB078, NCT03626012), and patient-specific *FUS* mutations [4] (Jacifusen, NCT04768972). Moreover, viral-based gene therapies are not far behind, and two patients have already been treated with AAV-vector-driven microRNA to lower *SOD1* levels [28]. Over the next decade, we hope one or more of these or other genetic medicines in development for ALS will be successful in directly intervening in the underlying disease etiology. If this is the case, the rNLS8 mice offer a unique tool to examine what would happen to the motor circuit thereafter.

Finally, most ALS patients do not have a known familial mutation that can be targeted. Therefore, we believe that discovering which axonal projections remain intact after the onset of TDP-43-triggered MN death can serve as a useful functional target to stabilize these synapses and encourage sprouting that could allow patients to maintain motor function for longer. Moreover, uncovering the molecular mechanisms that allow resistant MNs to cope with TDP-43 pathology could be beneficial for other diseases that feature TDP-43 as a major pathological hallmark in other cell types (for example, neurons in frontotemporal dementia). All neurodegenerative diseases feature some degree of selective disease resistance among neurons. Understanding the nature of selective resistance to neurodegenerative processes per se, and finding ways to encourage plasticity and neuronal compensation by resistant subtypes, could benefit patients in the whole class of devastating brain diseases.

## Supplementary Information

**Additional file 1**. Supplemental figures.
