# Supplementary material for: Slow motor neurons resist pathological TDP-43 and mediate motor recovery in the rNLS8 model of amyotrophic lateral sclerosis
Source: Acta Neuropathol Commun. 2022 May 14;10:75. doi: 10.1186/s40478-022-01373-0 (PMC9107273; doi:10.1186/s40478-022-01373-0)
Supplement: Supplementary file 1 — Additional file 1. Supplemental figures. [file 40478_2022_1373_MOESM1_ESM.docx]

Supplemental Figures for:

**Slow motor neurons resist pathological TDP-43 and mediate motor recovery in the rNLS8 model of amyotrophic lateral sclerosis**

Seong Kwon Hur^1,2^, Mandana Hunter^1,2^, Myrna A. Dominique^1,2^, Madona Farag^1,2^,
Dejania Cotton-Samuel^1,2^, Tahiyana Khan^1,2^, John Q. Trojanowski^1,2,3^, Krista J. Spiller^4,*^, Virginia M-Y Lee^1,2,3,*^

*^1^Center for Neurodegenerative Disease Research, Perelman School of Medicine at the University of Pennsylvania, Maloney Building, 3rd Floor, 3600 Spruce Street, Philadelphia, PA 19104-2676, USA;*

*^2^Department of Pathology and Laboratory Medicine, Perelman School of Medicine at the University of Pennsylvania, Philadelphia, PA, USA; ^3^Alzheimer’s Disease Research Center, Perelman School of Medicine at the University of Pennsylvania, Philadelphia, PA, USA; ^4^Janssen Research & Development, Neuroscience Therapeutic Area, Spring House, PA, USA.*

*Corresponding author email: [vmylee@upenn.edu](mailto:vmylee@upenn.edu) and [kspiller@its.jnj.com](mailto:kspiller@its.jnj.com)


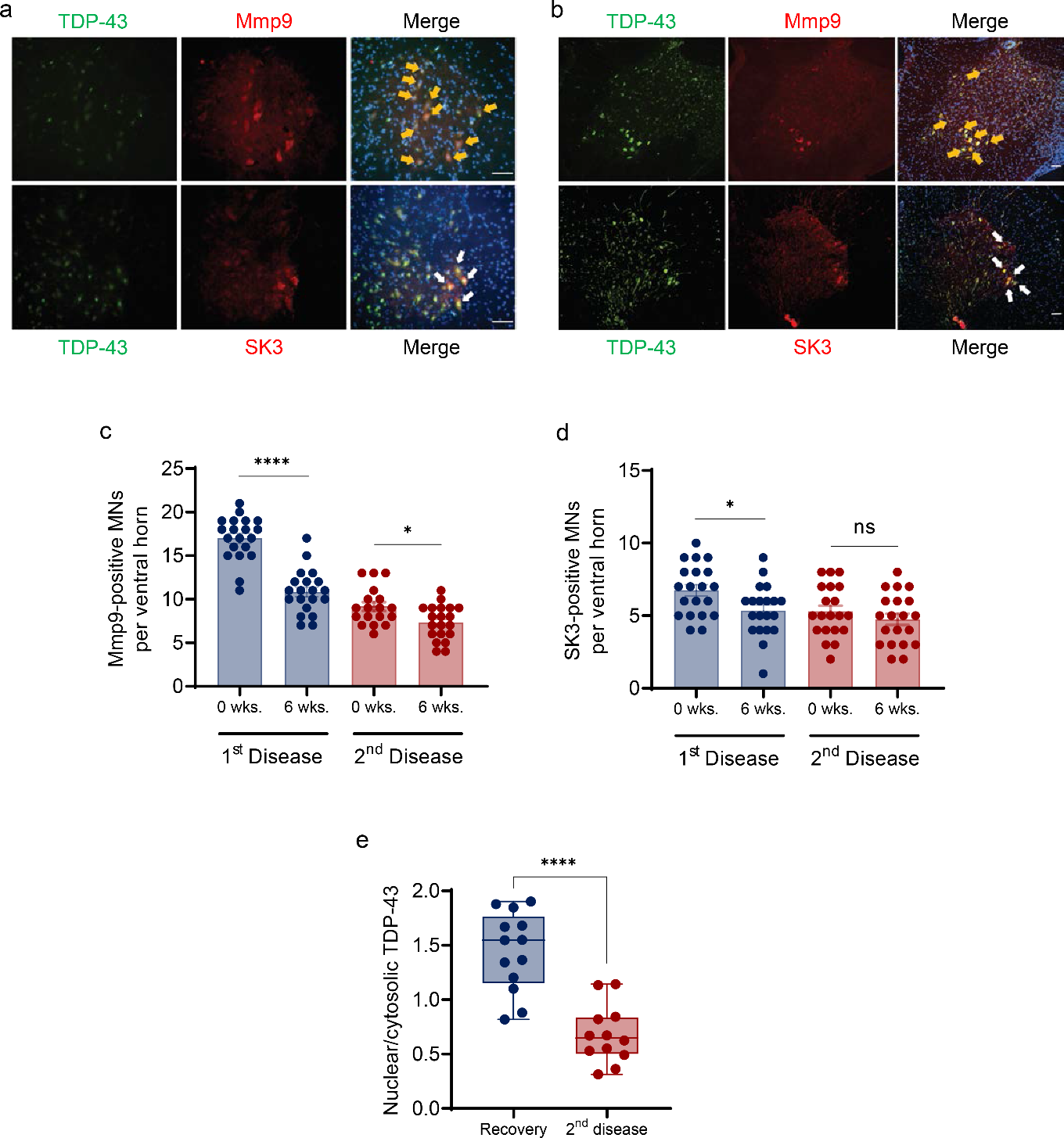


**Fig. S1** Slow MNs that resist TDP-43 pathology are recalcitrant to a second disease course despite high cytoplasmic expression of hTDP-43ΔNLS. **a, b** Representative immunofluorescence images of sections from the lumbar spinal cord of rNLS8 mice showing Mmp9-positive fast type MNs (red; top panels; marked by yellow arrows) and SK3-positive slow type MNs (red; bottom panels; marked by white arrows) showing low levels of TDP-43 (green) in mice recovering on Dox **a** or high levels of TDP-43 during the second disease course **b**. The antibody used for immunostaining is reactive for both human and murine TDP-43. **c, d** Quantitation of Mmp9-positive fast MNs (**c**; one-way ANOVA, ****, *p* < 0.0001; *, *p* = 0.0276) and SK3-positive slow MNs (**d**; one-way ANOVA, *, *p* = 0.0125) in the lumbar spinal cord of rNLS8 at baseline, after 6 wks. hTDP-43ΔNLS expression, during recovery on Dox or following a second 6-wk. instance of hTDP-43ΔNLS expression. Three mice were assayed per time point, with 20-22 ventral horn sections analyzed per animal. **e** Quantification of the relative immunofluorescence staining intensity for TDP-43 in the nucleus and cytoplasm of MNs in the lumbar region 4-6 of rNLS8 mice during recovery on Dox and after a second 6-wk. disease course. Unpaired t-test, ****, *p* < 0.0001; 30-35 cells scored per group


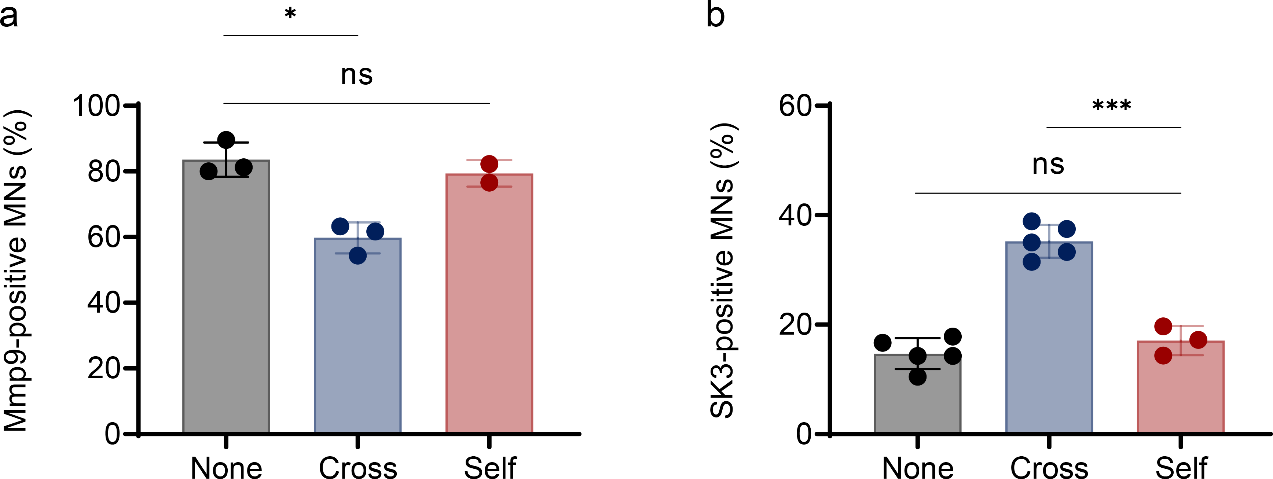


**Fig. S2** Cross-reinnervation surgery alters MN subpopulations in the TA muscle of rNLS8 mice. **a, b** Quantification of CTB-488-backfilled, re-innervated Mmp9-positive fast TA MNs (**a**; paired t-test, *, *p* = 0.0130; n. s., *p* = 0.4165) and SK3-positive slow TA MNs (**b**; paired t-test, ****, *p* < 0.0001; paired t-test, n. s., *p* = 0.2871) as a proportion of all CTB-488-positive TA MNs in the lumbar region 4-6 on the surgical and non-surgical sides of rNLS8 mice subjected to self-reinnervation (as a procedural control) or cross-reinnervation surgery. Three mice were analyzed per group 10 wks. after surgery, with 100-110 MNs scored per group

**
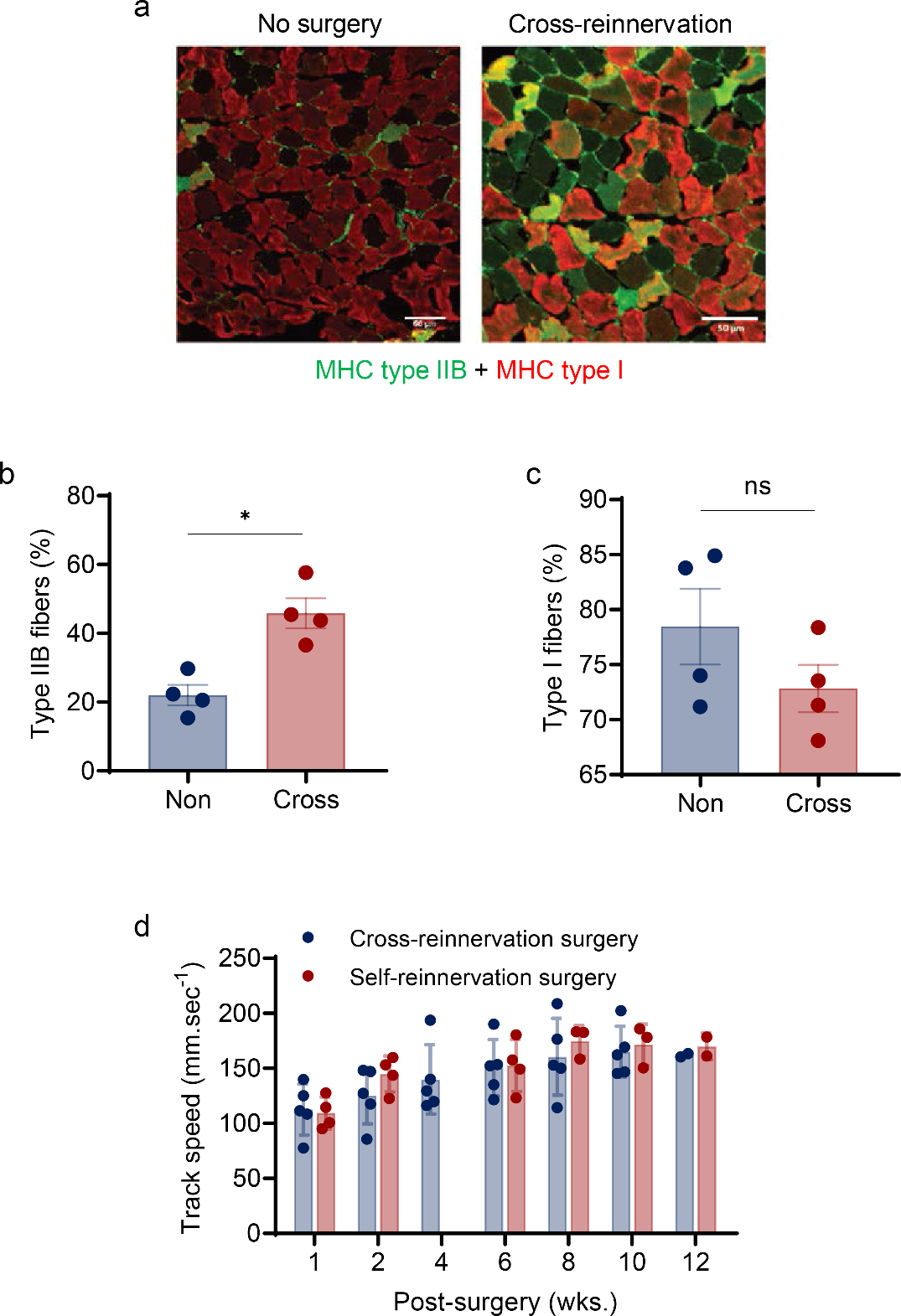
**

**Fig. S3** Soleus fiber type matches the substituted fast fatigable MNs after cross-reinnervation surgery in rNLS8 mice. **a** Representative images of fiber typing in the soleus muscle of rNLS8 mice performed 10 wks. after cross-reinnervation surgery. The left panel shows the soleus on the non-surgical side, whereas the right panel shows the soleus on the cross-reinnervation surgery side. Sections were immunostained for the slow fiber type marker myosin heavy chain (MHC) type I (BA-D5; green), and the fast fatigable fiber type marker MHC type IIB (BF-F3; red). Scale bar: 50 µm. **b, c** Quantification of MHC type IIB-positive fast-fatigable fibers (**b**; paired t-test, *, *p* = 0.0103) and MHC type I-positive slow fibers (**c**; paired t-test, n. s., *p* = 0.0785) as a proportion of all fibers on the surgical and non-surgical sides of rNLS8 mice subjected to cross-reinnervation surgery. Three animals were evaluated per group, with 500-700 fibers scored per group. **d** Instantaneous running speeds from TreadScan analysis of rNLS8 mice from 1 to 12 weeks after cross-reinnervation or self-reinnervation surgery. At each time point, 4-5 mice were assayed. No data were collected for the self-reinnervation surgery group at 4 wks.


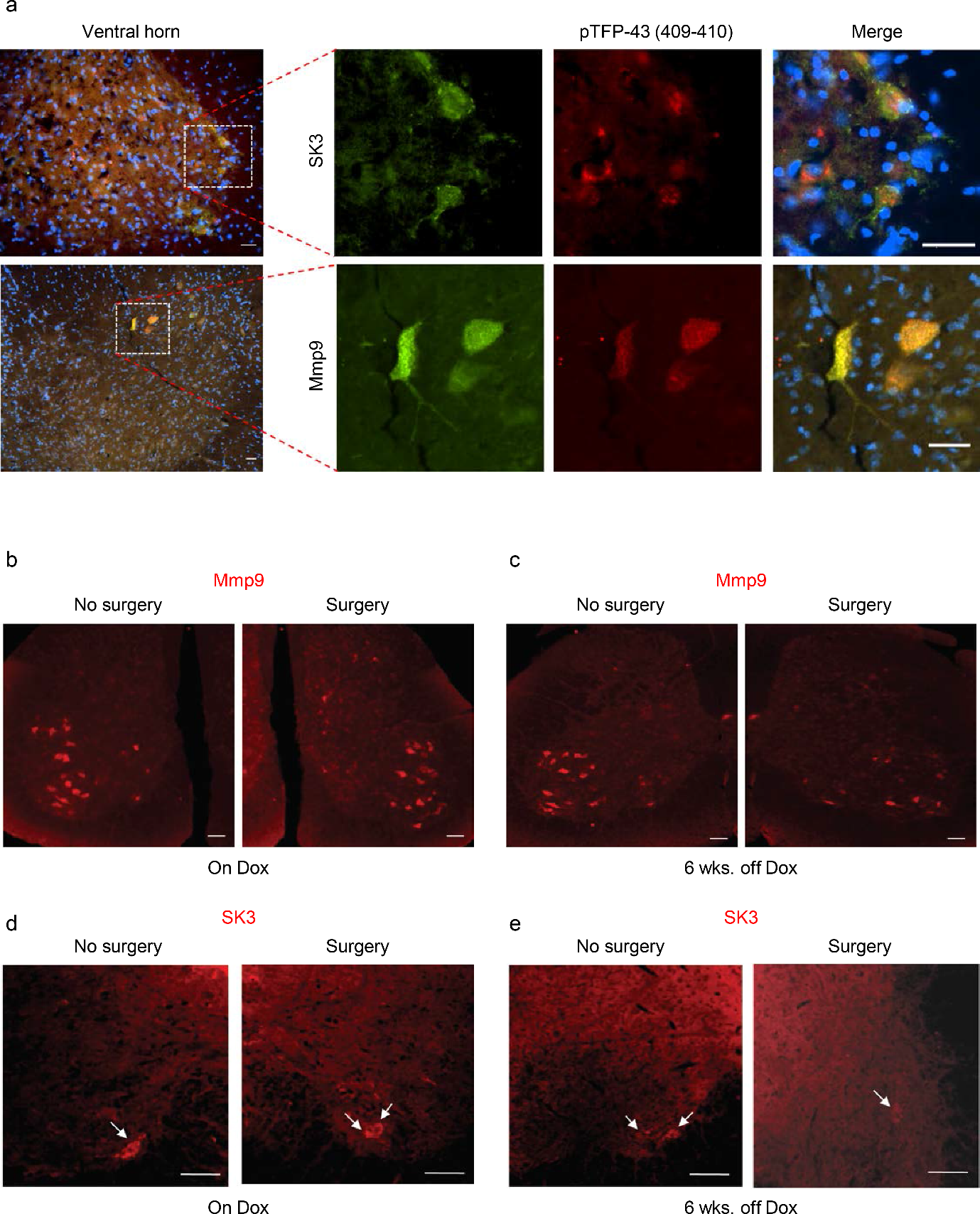


**Fig. S4** Fast motor units reinnervated by SK3-positive slow MNs remain resistant to pathological TDP-43 disease despite high expression of hTDP-43ΔNLS in both fast and slow MNs. **a** Representative fluorescence images showing immunostaining for SK3 (green; top panels), Mmp9 (green; bottom panels), and phosphorylated TDP-43 (S409-410; red) in the ventral horn on the surgical side of rNLS8 mice after 6 wks. hTDP-43ΔNLS expression. Scale bar: 50 µm. **b-e** Representative immunofluorescence staining for Mmp9 **b, c** and SK3 **d, e** corresponding to Fig. 4e-f in the main text and showing Mmp9-positive fast MNs in the lumbar regions 3-5 of rNLS8 mice on Dox **b** and 6 wks. off Dox **c**, or SK3-positive slow MNs (marked by white arrows) in mice on Dox **d** and 6 wks. off Dox **e**. Scale bar: 50 µm.
